# Effects of various pelvic floor muscle training forms and combined regimens on muscle strength, function, and recurrence rate in post-vaginal delivery stress urinary incontinence patients

**DOI:** 10.1016/j.clinsp.2026.101088

**Published:** 2026-09-05

**Authors:** YinJing Li, Chao Jin

**Affiliations:** a Department of Postpartum Rehabilitation, Zhejiang Shaoxing Shangyu Maternal and Child Health Hospital, Shaoxing City, Zhejiang Province, China; b Department of Gynaecology III, Shaoxing Maternity and Child Health Care Hospital, Shaoxing, Zhejiang, China

**Keywords:** Stress urinary incontinence, Training intensity, Modality, Comparative effectiveness

## Abstract

**Objective:**

To investigate the effects of focused PFMT (Kegel exercises), generalized PFMT, and high-intensity combined PFMT on Pelvic Floor Muscle (PFM) strength, functional recovery, and Stress Urinary Incontinence (SUI) recurrence in women with Postpartum Stress Urinary Incontinence (PSUI).

**Methods:**

Ninety-nine PSUI patients were randomly divided into three groups: Focused PFMT group (precise gluteal contractions), Generalized PFMT group (comprehensive PFM training), and High-Intensity Combined group (integrating both). After 4-weeks of treatment, assessments included PFM strength grading, urodynamic parameters (including Maximum Urethral Closure Pressure [MUCP], Maximum Urethral Pressure [MUP], Functional Urethral Length [FUL], Bladder Capacity [BC], and maximum flow rate [Qmax]), Urinary Incontinence Impact Questionnaire (IIQ-7) scores, 1-hour pad test leakage volume, and serum Estradiol (E₂), Follicle-Stimulating Hormone (FSH), and Relaxin (RLX) levels. Recurrence rates were assessed at 3- and 6-months.

**Results:**

All groups showed improved PFM strength after treatment, with the Combined Group exhibiting a significantly higher proportion of Grade IV‒V strength. Urodynamic parameters, IIQ-7 scores and leakage volume improved in all groups, with the Combined Group achieving the most significant improvements. The Combined Group showed superior clinical efficacy and a significantly lower 6-month recurrence rate.

**Conclusion:**

Combining focused and Generalized PFMT effectively enhances pelvic muscle strength, improves urethral and bladder function, and reduces SUI recurrence. The efficacy of PFMT is closely related to training intensity, suggesting that a high-intensity protocol combining focused and comprehensive exercises may be the optimal choice for managing postpartum SUI. The therapeutic benefits may be associated with hormonal modulation.

## Highlights

• Kegel + PFMT (Group C) showed the greatest improvement in pelvic muscle strength, urodynamic parameters, and symptom relief.
• Group C had significantly lower SUI recurrence rates at 6-month follow-up, highlighting long-term benefits of combined training.
• Therapy correlated with improved Estrogen (E_2_) and Relaxin (RLX) levels, suggesting hormonal modulation may contribute to efficacy.
• Provides evidence for combined pelvic floor rehabilitation as a comprehensive approach for postpartum SUI management.

## Introduction

Postpartum Stress Urinary Incontinence (PSUI) refers to the involuntary leakage of urine from the urethral orifice during sudden increases in abdominal pressure caused by sneezing, coughing, laughing, exercise, labor, or straining during defecation.^1^ PSUI significantly impacts patients’ quality of life and mental health. It may result from pregnancy- and delivery-induced damage to pelvic floor structures, weakened pelvic muscle strength, ligament laxity, and hormone-related urethral dysfunction.^2^ PSUI is a common pelvic floor disorder. The prevailing theory suggests that disruption of bladder/urethral supportive connective tissues and weakening of pelvic floor, bladder neck, and urethral sphincter muscles reduce urethral closure pressure, functionally contributing to PSUI. Studies report that 25%–55% of postpartum women experience urinary incontinence, with initial onset typically occurring after vaginal delivery (3.7%–19%).^3^^,^^4^ A Chinese survey of 19,024 SUI patients revealed a 30.9% incidence.^5^ Notably, among women reporting SUI symptoms at 3-months postpartum, 24% and 37.9% still exhibited persistent symptoms at 6- and 12-years postpartum, respectively,^6^ indicating the condition’s chronicity without intervention.

Current interventions include surgical and non-surgical treatments. Surgical approaches (e.g., anatomical reconstruction or transvaginal mesh) face limitations such as high recurrence (nearly one-third of cases require reoperation),^7^ cost, and infection risks.^8^ Non-surgical treatment is the first-line management for PFD,^9^ with Pelvic Floor Muscle Training (PFMT) being a core non-surgical approach. PFMT primarily involves training through pelvic floor muscle contractions, thereby increasing urethral resistance and pelvic floor support tension, ultimately enhancing urinary control capability.^10^ It is important to clarify that Generalized PFMT and focused PFMT (Kegel exercises) are two common forms of PFMT implementation. Generalized PFMT involves exercises such as anal contractions, leg movements, and sit-ups, targeting the pelvic floor muscles and core muscle groups, and focuses on the holistic recovery of muscle group function. However, this comprehensive training lacks specificity and may fail to achieve ideal results. In contrast, Focused PFMT (Kegel exercises) centers on the precise contraction of the pubococcygeus muscle, emphasizing targeted exercise for specific pelvic floor muscle groups.^11^ This therapy was first introduced in 1970 by Dr. Arnold Kegel, who invented it as a method for exercising the strength of the pubococcygeus muscle. Currently, it has been proven suitable as a routine PFM exercise for postpartum women, showing good effects in improving various types of urinary incontinence, vaginitis, and puerperium symptoms. Although the effectiveness of PFMT has been confirmed by numerous studies,^12^ current research remains contentious. Most studies focus on the efficacy differences between PFMT and non-PFMT interventions, lacking direct comparisons between different forms of PFMT implementation. Some studies erroneously classify the combination of focused Kegel exercises and standard comprehensive training simply as a combination of two therapies, failing to clarify whether the high-intensity training formed by superimposing these 2 p.m. forms can further enhance therapeutic efficacy.

Based on the aforementioned research gaps, this study designs three different forms of PFMT protocols, aiming to clarify the impact of PFMT intensity and implementation modality on the efficacy for postpartum PSUI patients. This study hypothesizes that high-intensity combined PFMT, due to its higher total training intensity and its simultaneous possession of the muscle group specificity of focused training and the multi-muscle group coverage advantage of comprehensive training, will demonstrate superior efficacy in improving pelvic floor muscle strength, alleviating urinary incontinence symptoms, and preventing recurrence compared to single-form PFMT. Furthermore, focused PFMT, owing to its more precise movements, may yield better outcomes than comprehensive PFMT. Through systematic experimental design and data analysis, the study seeks to assess the combined therapy's efficacy and provide more robust clinical evidence.

## Materials and methods

### Sample collection

A prospective study was conducted on 126 patients with PSUI who visited the gynecological outpatient clinic and female rehabilitation specialty department of Zhejiang Shaoxing Shangyu Maternal and Child Health Hospital between June 2023 and July 2024. Participants were randomly assigned using a random number table to three groups: the Focused PFMT group, the Generalized PFMT group, and the High-Intensity Combined PFMT group.

Inclusion Criteria: 1) Patients meeting clinical diagnostic criteria; 2) Patients aged >18-years, primiparous, and naturally conceived; 3) Patients with singleton pregnancy; 4) Patients with full-term delivery (gestational age ≥37-weeks); 5) Postpartum period ≥12-weeks; 6) Patients voluntarily participating in the study with agreeing to follow-up, and signing informed consent; 7) A score of ≥12 on the Kegel Exercise Adherence Questionnaire and completion of at least 80% of the prescribed training sessions were required for a participant to be considered adherent.

Exclusion Criteria: 1) Patients not meeting the above diagnostic criteria; 2) Patients receiving SUI medication within the past 3-months; 3) Patients with urinary frequency/urgency or concurrent urinary tract infections; 4) Patients meeting surgical indications for SUI; 5) Patients with severe cardiovascular, cerebrovascular, renal, or coagulation disorders; history of pelvic floor or incontinence surgery; diabetes; cauda equina or spinal cord lesions; or neurological diseases; 6) Patients with other types of urinary incontinence; 7) Patients with mobility limitations (e.g., inability to climb stairs). All participants were informed that they were taking part in a postpartum pelvic floor rehabilitation study involving three different training regimens, all of which were clinically validated as effective; however, they were not informed of the specific differences between the group assignments.

### Sample size calculation

The pre-trial data indicated that the proportion of patients achieving pelvic floor muscle strength grades IV‒V after 4-weeks of treatment was a short-term efficacy indicator showing more significant intergroup differences. This proportion was used as the primary outcome measure for sample size calculation. With the significance level (α) set at 0.05 (two-tailed) and the target statistical power (1-β) set at ≥ 80%, a sample size of 33 participants per group was determined. This allocation ensured an effective sample size of ≥ 30 participants per group. Validation using G*Power 3.1 software showed that the statistical power for the primary outcome with the above parameters reached 91.2%, thereby satisfying the requirement of ≥ 80%.

### Diagnostic criteria

The diagnosis was established according to the 2017 “Guidelines for Diagnosis and Treatment of Female Stress Urinary Incontinence” issued by the Pelvic Floor Disorders Group of the Chinese Society of Obstetrics and Gynecology. Diagnostic requirements included: 1) Symptomatic presentation of involuntary urine leakage from the urethral orifice during sudden intra-abdominal pressure elevation (e.g., laughing, coughing, exercise, sneezing); 2) Positive stress provocation test ‒ performed with the patient in a supine position with a full bladder, observing urethral leakage during forceful coughing, with repetition in standing position if negative initially; 3) Absence of concomitant urinary frequency or urgency symptoms.

### Therapeutic methods and supervision

Nurses first assessed patients to evaluate their understanding of the pelvic floor muscles. Standardized instructions were used to educate all patients, ensuring they could identify and understand the relevant muscle groups. A senior nurse (at or above the supervisory level) then demonstrated the exercises, provided guidance, and monitored the accuracy of the patients' movements. One week before the formal trial, a single therapist instructed all patients on the correct method for contracting the pelvic floor muscles until proficiency was achieved. The proficiency criteria were visible observation of significant pelvic floor muscle contraction and ultrasound confirmation of bladder base elevation. Medical staff also taught patients how to perform and self-assess the pelvic floor muscle exercises at home. Patients performed their exercises at home using an audio-guided training version provided by our hospital. They were taught to selectively control their pelvic floor muscles while keeping other muscles relaxed. Patients were instructed to wear disposable gloves, insert the middle and index fingers into the vagina, and then perform an anal contraction. A sensation of pressure around the fingers indicated a proper pelvic floor muscle contraction; the absence of this sensation indicated an improper contraction. An intravaginal examination was conducted every two weeks to adjust the exercise regimen as needed. Follow-up and reminders were provided via telephone by medical staff. Caregivers supervised the patients' daily training and encouraged them to record their completion in a logbook.

The Generalized PFMT group performed a comprehensive regimen of anal, leg, and sit-up exercises. The specific protocol included: 1) Anal contraction exercises: Patients lay in the supine position with an empty bladder. They were guided to voluntarily contract their pelvic floor muscles without engaging the abdominal, gluteal, or lower limb muscles, mimicking the action of suppressing defecation to perform a lifting motion, feeling the perianal skin retract inwards. Patients placed their hands lightly on the abdomen to confirm no movement. Each contraction was held for 5 s, followed by a slow relaxation for 3 s, alternating continuously. This was performed for 10-minutes per session, once daily. 2) Leg exercises: Patients lay supine and performed regular, rapid extensions and flexions of both legs for 10 min per session, once daily. 3) Sit-up exercises: Patients were positioned in a 45° semi-Fowler's position using an adjustable bed. Their legs were stabilized with hips and knees flexed, feet flat on the bed. With hands in a Bobath handclasp, patients independently performed sit-ups, ensuring abdominal muscles remained engaged throughout the movement. This was performed for 10-minutes per session, once daily.

The Focused PFMT group performed Kegel exercises. Before exercising, patients emptied their bladder and assumed a supine position, relaxing the whole body, concentrating mentally, and breathing deeply and slowly with knees naturally bent and hands resting at their sides. During inhalation, the muscles of the perineum (including the anal and urethral sphincters) were contracted upwards. Guidance was provided in two steps: first, patients imagined the action of interrupting urination (feeling the muscles around the urethra tighten inwards), then superimposed the action of suppressing defecation (contracting the anus inwards and upwards). The contraction was held for 5-seconds, followed by slow relaxation during exhalation, repeating the cycle after a 5-second rest. Patients were instructed to keep other parts of the body relaxed during the exercise. Each session lasted 30-minutes, performed once daily.

The High-Intensity Combined Group performed a regimen integrating both the Generalized PFMT and Focused PFMT protocols. The training methods and rhythms were identical to those described above. All three groups underwent continuous treatment for 4 weeks. For the detailed training plan, see Supplementary Table 1.

### Assessment of training adherence and tolerance

The Kegel Exercise Adherence Questionnaire was specifically designed to assess adherence to Kegel exercises and was developed by researcher Chen et al. in Taiwan. The questionnaire primarily consists of three core sections: 1) The daily time commitment required for pelvic floor muscle training; 2) The average number of contractions performed per training session; and 3) A visual analog scale for adherence to pelvic floor muscle training, which requires selecting a number from 0 to 10 that most accurately reflects the level of adherence. A total score is calculated by combining the scores from these three sections to evaluate the patient’s adherence to pelvic floor muscle training. The total score ranges from 2 to 21, with a higher score indicating better adherence. After undergoing reliability analysis and validity testing, the questionnaire has been confirmed to possess good reliability and validity, making it suitable for application in clinical settings. The Cronbach’s α coefficient for this scale is 0.81.

### Outcome measures

#### Clinical data collection

Demographic and clinical characteristics, including age, Body Mass Index (BMI), and pregnancy-related parameters, were recorded for all participants. All patient medical records and test data were documented using anonymous identification codes, with any information potentially indicating group allocation removed.

### Urodynamic assessment

Maximum Urethral Closure Pressure (MUCP), Maximum Urethral Pressure (MUP), Functional Urethral Length (FUL), Bladder Capacity (BC), and maximum flow rate (Qmax) were measured pre- and post-treatment using a urodynamic testing system (Nidoc 970A+, Weixin Medical) to evaluate urethral sphincter function and bladder storage/voiding capacity.

### Pelvic floor pressure measurement

Vaginal Squeeze Pressure (VSP) and Vaginal Resting Pressure (VRP) were assessed using a Neuromuscular electrical stimulator (—DS-B, Jiangsu Furuai Technology Co., Ltd.) at baseline and post-treatment. VSP reflects active contraction pressure while VRP indicates resting tone of pelvic floor muscles.^13^ The technician, blinded to the participants' assigned training regimens, recorded data strictly according to the instrument’s operating manual, and all data files were named using anonymous identification codes.

### Pelvic muscle strength grading

Muscle strength was evaluated using the standardized perineometry classification system.^14^ The assessor, wearing gloves during the procedure, determined the grade solely based on the duration of the muscle contraction. Pelvic floor muscle strength was graded on a 6-level scale: Grade 0: No contraction (0 s duration); Grade I: 1 s sustained contraction; Grade II: 2 s sustained contraction; Grade III: 3 s sustained contraction; Grade IV: 4 s sustained contraction; Grade V: ≥ 5 s sustained contraction. Adequate strength rate (%) was calculated as: (Number of Grade IV + V cases)/Total cases × 100%.

### 1-hour pad weight test

Pad weight measurements were conducted before treatment and 1-day post-treatment. Participants consumed 500 mL of water 10-minutes prior to testing, followed by 30-minutes bed rest, 10-minutes stair climbing, 10-minutes walking, 2-minutes sitting-standing, 1 minute stationary running, 1 minute hand washing, and 30 forceful coughs. The pre-weighed pads were subsequently measured for urine leakage volume.

### Urinary incontinence scoring

The Urinary Incontinence Impact Questionnaire (IIQ-7) was administered pre-treatment and 1-day post-treatment to assess symptom severity.^15^^,^^16^ The scale (0‒21 points) evaluates three domains: leakage frequency (0–5 points), leakage volume (0–6 points), and quality-of-life impact (0–10 points), with higher scores indicating greater severity.

### Hormonal and serum analysis

Venous blood samples (5 mL) were collected pre- and post-treatment. Serum Estradiol (E_2_) and Follicle-Stimulating Hormone (FSH) levels were quantified using chemiluminescent immunoassay (Roche Diagnostics kits) per manufacturer protocols.

### Ultrasonic assessment of bladder anatomical position

A transperineal two-dimensional ultrasound system (E6, Voluson) was used with a preset pelvic floor imaging mode. The depth was adjusted to 8‒10 cm and the gain was uniformly set to 60 dB to ensure consistent imaging conditions across different patients. All examinations were performed by the same physician with over five years of experience in pelvic floor ultrasound. After the patient's bladder was filled to 150‒200 mL, the patient was placed in the lithotomy position with the hips slightly elevated (using a 5 cm thick sterile pad), legs abducted to shoulder width, and the perineum exposed. The probe was placed on the perineal area between the external urethral orifice and the anus. Static measurements were taken during quiet breathing, recording the vertical distance from the bladder neck to the inferior edge of the pubic symphysis (BN-PU) and the angle (β-angle) between the urethral axis and the tangent line of the bladder base. For dynamic measurements, patients were instructed to perform a slow Valsalva maneuver (holding breath after deep inspiration, with a slow increase in abdominal pressure). The maneuver was repeated three times, and the maximum downward displacement distance of the Bladder Neck (BND) was recorded, calculated as the difference between the BN-PU value at quiet breathing and the BN-PU value during the Valsalva maneuver. The efficacy criteria were defined as follows: Markedly Effective: Static BN-PU value ≥ 30 mm and β-angle between 90° and 120°, with dynamic BND < 15 mm; Effective: The static BN-PU value increased by ≥ 3 mm compared to pre-treatment, or the dynamic BND decreased by ≥ 3 mm compared to pre-treatment; Ineffective: The static BN-PU value increased by < 3 mm or decreased compared to pre-treatment, and the dynamic BND decreased by < 3 mm or increased compared to pre-treatment.

### Clinical efficacy evaluation

Based on the degree of symptomatic improvement, the pad test results, and the ultrasonic assessment of bladder anatomical position, clinical efficacy was explicitly categorized into the following three grades:

Markedly Effective: The number of leakage episodes during increased abdominal pressure decreased by ≥ 70% compared to pre-treatment (based on patient-recorded daily leakage counts); the 1-hour pad test leakage volume was < 1 mL (weight difference < 1 g); and transperineal ultrasound assessment showed the bladder neck position was restored to the normal range.

Effective: The number of leakage episodes decreased by ≥ 50% but < 70% compared to pre-treatment; the 1-hour pad test leakage volume was 1‒5 mL (weight difference 1‒5 g); and transperineal ultrasound assessment showed an improvement in bladder neck position of ≥ 3 mm compared to pre-treatment.

Ineffective: The number of leakage episodes decreased by < 50% compared to pre-treatment; the 1-hour pad test leakage volume was > 5 mL (weight difference > 5 g); and the ultrasonic assessment of bladder anatomical position showed no improvement or worsening (improvement < 3 mm).

The overall response rate was calculated as: (Number of Markedly Effective cases + Number of Effective cases) / Total cases × 100%.

All efficacy judgments were performed by one gynecological ultrasonographer and one pelvic floor rehabilitation physician, both with over five years of experience in pelvic floor ultrasound. The assessors were blinded to the patients’ group assignments.

### Recurrence rate assessment

Baseline enrollment was set at 12-weeks postpartum. All subjects underwent a 4-week intervention after enrollment. All groups underwent follow-up assessments at 3- and 6-months post-treatment to document SUI recurrence.

### Statistical analysis

All analyses were performed using Statistical Package for the Social Sciences version 22.0. Data normality was assessed using the Shapiro-Wilk test. Continuous data were expressed as either mean ± Standard Deviation (SD) for parametric distributions or median and interquartile range for non-parametric distributions. Baseline characteristics were compared between groups using one-way analysis of variance for normally distributed continuous variables with homogeneity of variance. The Pearson chi-square test was employed for overall comparisons between the groups. When significant differences were detected (p < 0.05), post-hoc analyses were conducted using either Tukey’s HSD test for continuous variables or Bonferroni correction for categorical variables. Pelvic floor muscle strength, being ordinal data (Grade 0‒V), was compared for baseline distribution across groups using the Kruskal-Wallis H test. For repeated measures data (e.g., pre- vs. post-treatment comparisons), a two-way ANOVA was employed for assessment, followed by Tukey's HSD test for post-hoc multiple comparisons. Within-group comparisons of pelvic floor muscle strength (ordinal data) before and after treatment were performed using the Wilcoxon signed-rank test, with the Type I error rate for multiple comparisons controlled via Bonferroni correction. An independent assessment team consisting of three gynecological rehabilitation specialists, who were not involved in training guidance and were blinded to group assignments and specific training protocols, evaluated all patient outcomes. An anonymous questionnaire survey revealed that only 28.3% (28/99) of patients correctly guessed their group assignment, and the training instructors correctly guessed the assignments for 31.2% (10/32) of the patients they supervised. The correct guess rates for both subjects and instructors were below 35%, indicating successful implementation of blinding in this study. The significance level for all statistical analyses was set at 0.05, and marginal p-values (e.g., p = 0.05) were explicitly defined as statistically non-significant.

## Results

### Assessment of treatment adherence, tolerance, and comparison of baseline data among the three patient groups

A total of 126 patients were screened for this study, of whom 27 were excluded for the following reasons: 4 were lost to follow-up, 5 refused to continue participation during the training period, and 16 were excluded due to failure to complete the exercises for more than one consecutive week. The primary adherence difficulty was related to completing all daily exercises. In the first week of training, over 10% of patients in both the Focused PFMT group and the Generalized PFMT group reported not completing the exercises on schedule, while over 20% of patients in the High-Intensity Combined Group, due to the higher training intensity, reported the same issue. A similar trend persisted at the fourth week. Subjective tolerance was assessed throughout the study period. In the first week, no patients in the Focused PFMT or Generalized PFMT groups reported discomfort, whereas 20% of patients in the High-Intensity Combined Group reported some discomfort. After active encouragement for patients to persist with the training, 11% of patients in the High-Intensity Combined Group still reported mild discomfort by the fourth week. No study withdrawals due to adverse reactions occurred.

The systematic evaluation of clinical outcomes revealed no statistically significant differences (p > 0.05) among the three PSUI groups regarding age, pre-pregnancy BMI, BMI at 34 gestational weeks, education level, gestational age at delivery, or neonatal birth weight (Table 1), confirming balanced baseline characteristics.

**Table 1:** Comparative analysis of baseline characteristics among the three groups.

Parameter	Focused PFMT Group (n = 33)	Generalized PFMT Group (n = 33)	High-Intensity Combined Group (n = 33)	p-value
Age (year)	30.59 ± 1.91	30.17 ± 1.80	29.90 ± 1.65	0.30
Pre-gravid BMI (kg/m^2^)	20.60 ± 1.92	19.97 ± 1.79	19.87 ± 1.98	0.25
BMI at 34-weeks’ gestation (kg/m^2^)	23.87 ± 1.76	23.33 ± 1.69	23.00 ± 1.94	0.14
Education				0.89
Below Bachelor	10 (30.30%)	9 (27.27%)	8 (24.24%)	
Bachelor	17 (51.52%)	15 (45.45%)	18 (54.55%)	
Above Bachelor	6 (18.18%)	9 (27.27%)	7 (21.21%)	
Gestational age at delivery (weeks)	39.57 ± 0.44	39.50 ± 0.46	39.49 ± 0.49	0.72
Neonatal birth weight (g)	3378.36 ± 430.90	3276.57 ± 464.64	3294.51 ± 490.74	0.64
Compliance Questionnaire Score	16.20 ± 2.10	15.80 ± 1.90	16.50 ± 2.30	0.40
Training Completion Rate	29 (87.88%)	28 (84.85%)	26 (78.88%)	0.70
Reported Discomfort	3 (9.09%)	2 (6.06%)	6 (18.18%)	0.37
Prior Knowledge of Kegel Exercises				0.11
Yes	16 (48.48%)	22 (66.67%)	24 (72.73%)	
No	17 (51.52%)	11 (33.33%)	9 (27.27%)	

Note: Normally distributed continuous data are presented as mean ± Standard Deviation (SD). Intergroup comparisons were performed using one-way analysis of variance. Categorical data are presented as n (%). Chi-Square test was used for categorical variables. p < 0.05 was considered statistically significant.

BMI, Body Mass Index.

### Pelvic floor parameters before and after treatment

**Fig. 1:**
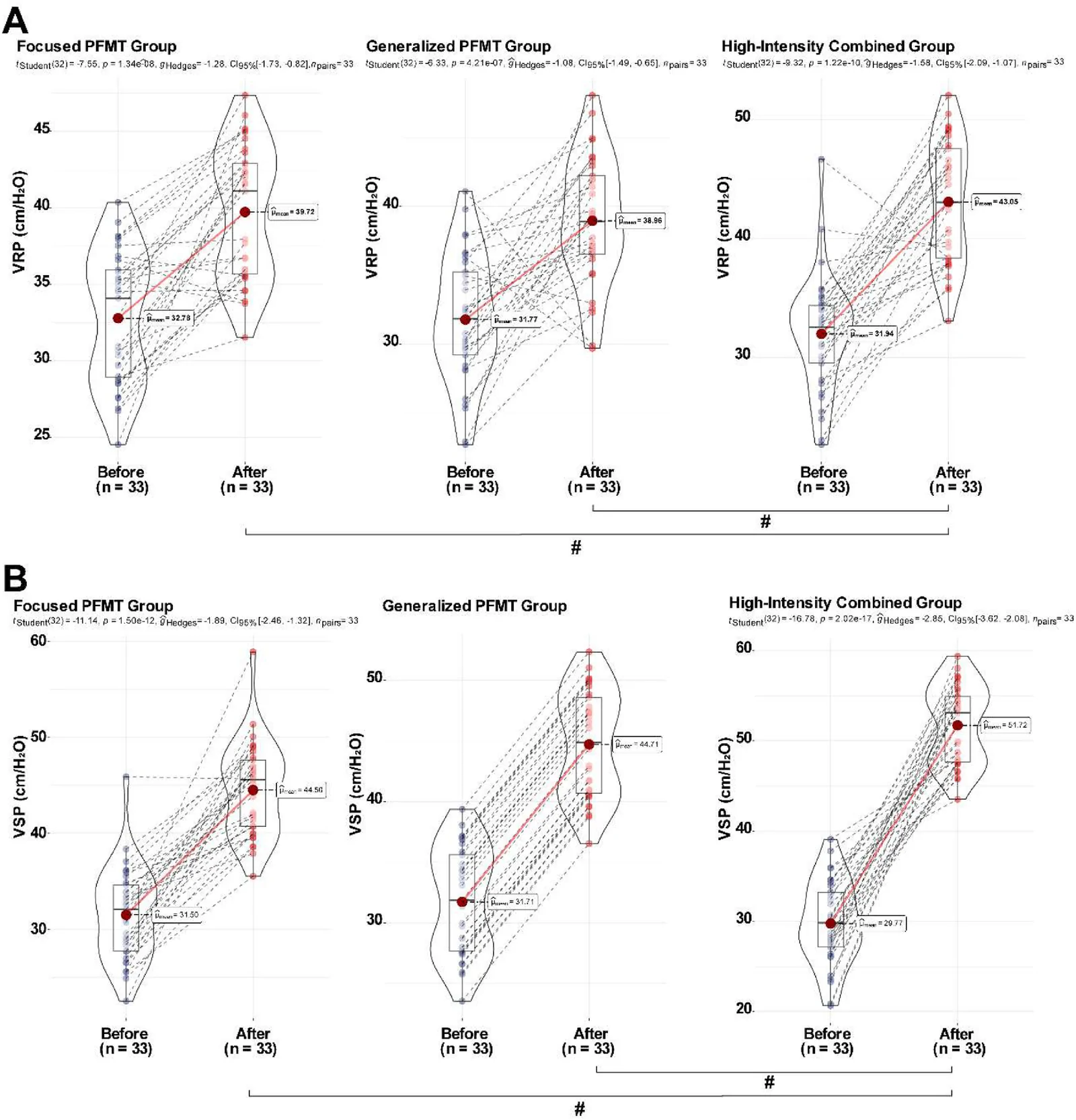
**Comparison of VSP and VRP among three PSUI patient groups before and after treatment.** (A) Comparison of VSP among the three groups before and after treatment; (B) Comparison of VRP among the three groups before and after treatment. Error bars represent mean ± SD; scatter points represent raw data from individual patients, and box plots represent the median and interquartile range (Q1‒Q3). ** Intra-group pre-post comparisons were performed using the Wilcoxon signed-rank test, * p < 0.05; comparisons of post-treatment changes between groups were performed using the Wilcoxon rank-sum test with Bonferroni correction, ^#^ p < 0.05. ** Focused PFMT group (n = 33); Generalized PFMT group (n = 33); High-Intensity Combined Group (n = 33).

As shown in Table 2, pretreatment pelvic muscle strength distributions were comparable across groups (p = 0.91). Post-treatment assessment revealed significant differences in muscle strength grade distribution among the three groups (p < 0.01). Ordinal regression analysis demonstrated that the improvement in muscle strength was significantly greater in the High-Intensity Combined Group than in the Focused PFMT group (β = 0.87, 95% CI 0.21–1.53, p = 0.03) and the Generalized PFMT group (β = 0.92, 95% CI 0.25–1.59, p = 0.01). Pressure measurements indicated marked improvements in VRP and VSP across all groups, with the High-Intensity Combined Group exhibiting the most pronounced enhancements (Fig. 1A‒B).

**Table 2:** Comparison of pelvic floor muscle strength before and after training among the three groups.

Group	Before Training			After Training		
	Grade 0‒I	Grade II‒III	Grade IV‒V	Grade 0‒I	Grade II‒III	Grade IV‒V
**Focused PFMT Group (A)**	12 (36.36%)	16 (45.45%)	5 (15.15%)	10 (30.30%)	14 (42.42%)	9 (27.27%)
**Generalized PFMT Group (B)**	15 (45.45%)	15 (45.45%)	3 (9.09%)	11 (33.33%)	13 (39.39%)	9 (27.27%)
**High-Intensity Combined Group (C)**	13 (39.39%)	15 (45.45%)	5 (15.15%)	7 (21.21%)	12 (36.36%)	15 (42.42%)
adj.*P*_Group A/Group B_			1.00			1.00
adj.*P*_Group A/Group C_			1.00			0.03
adj.*P*_Group B/Group C_			1.00			0.01
p-value			0.91			0.00

Note: Categorical data are presented as n (%). Overall differences were analyzed using Pearson’s Chi-Square test. When significant differences were found, post-hoc comparisons were performed using Bonferroni correction. Adjusted p-values < 0.05 were considered statistically significant.

### Urodynamic parameter analysis

**Fig. 2:**
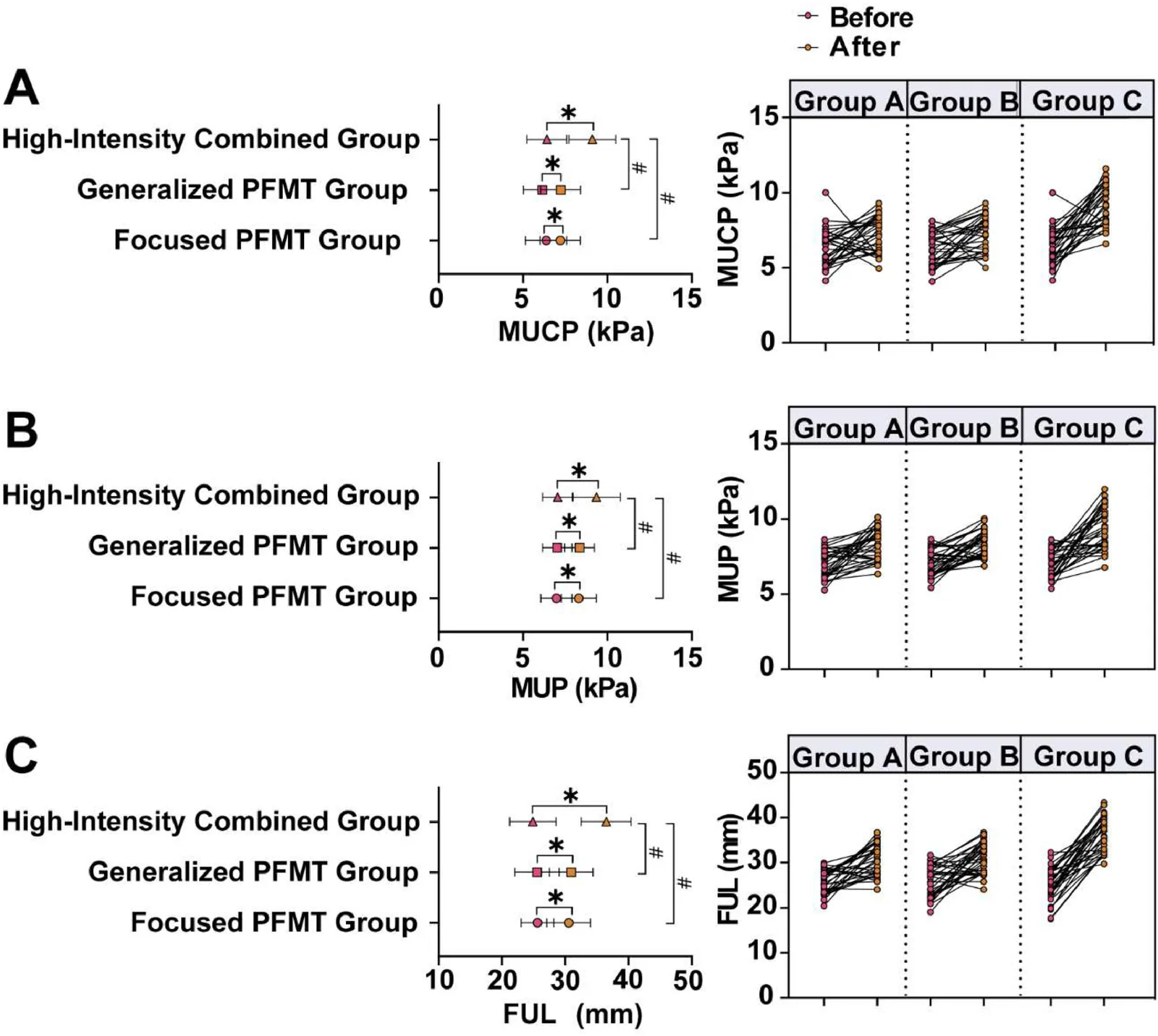
**Comparison of urodynamic parameters among three groups.** (A‒C) Comparison of MUCP, MUP, and FUL indices among patients before and after treatment. Error bars represent mean ± SD; scatter points represent raw data from individual patients. **Intra-group pre-post comparisons were performed using the Wilcoxon signed-rank test, *p < 0.05; post-treatment data comparisons between groups were performed using Tukey’s HSD test, ^#^p < 0.05. Notes: Group A, Focused PFMT group; Group B, Generalized PFMT group; Group C, High-Intensity Combined Group.

**Fig. 3:**
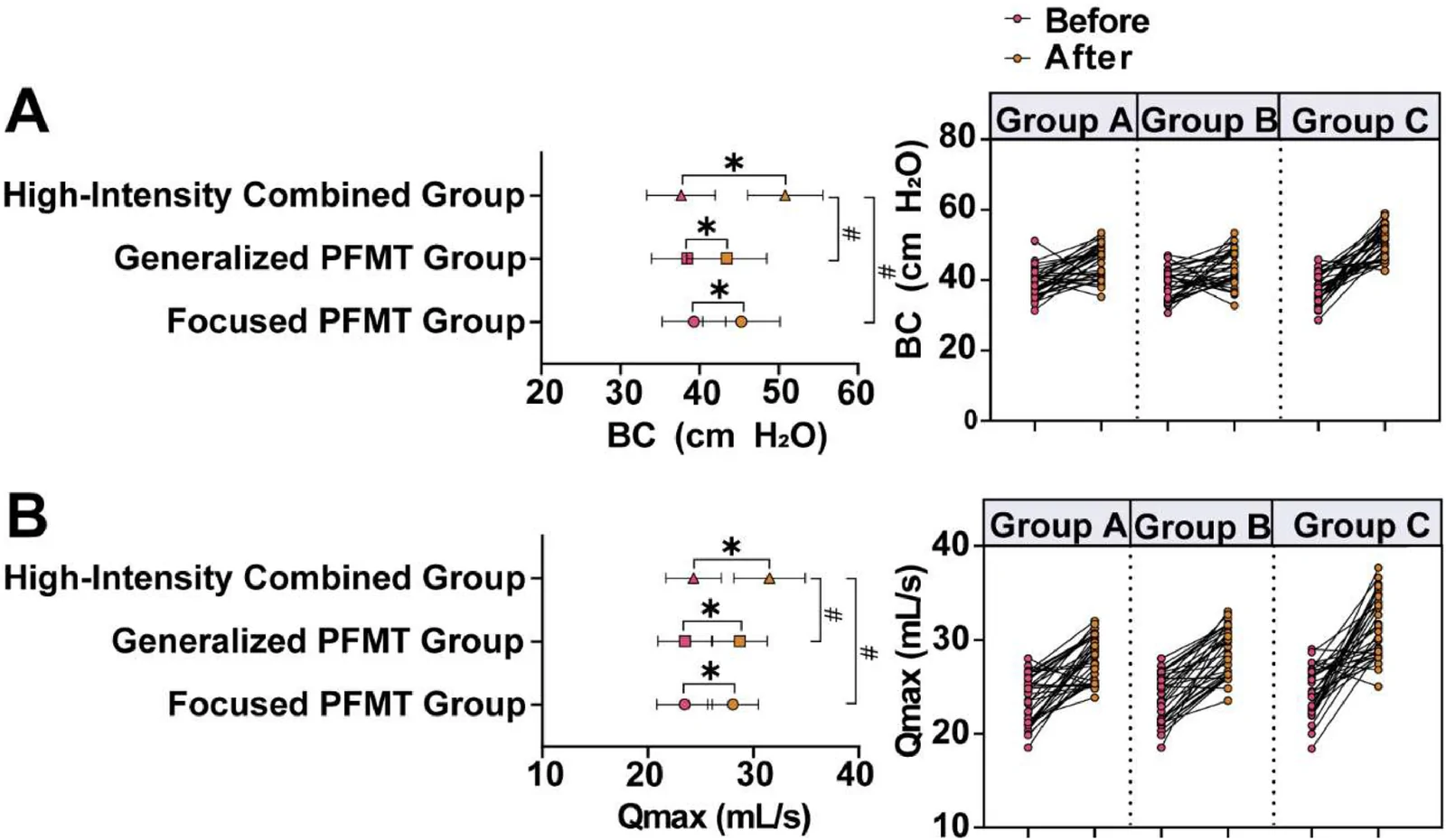
**Comparison of BC and Qmax levels among the three groups.** (A‒B) Comparison of BC and Qmax among patients before and after treatment. Error bars represent mean ± SD; scatter points represent raw data from individual patients. ** Intra-group pre-post comparisons were performed using the Wilcoxon signed-rank test, * p < 0.05; post-treatment data comparisons between groups were performed using Tukey’s HSD test, ^#^ p < 0.05. Notes: Group A, Focused PFMT group; Group B, Generalized PFMT group; Group C, High-Intensity Combined Group.

Pretreatment urodynamic measurements showed no intergroup differences (all p > 0.05). Post-intervention, all groups exhibited increased MUCP, MUP, FUL, BC, and Qmax values. The regimen combining Focused PFMT and Generalized PFMT achieved the most substantial improvements in MUCP and MUP, and also demonstrated the greatest increase in FUL (Fig. 2A‒C). Furthermore, both BC and Qmax were significantly increased compared to pre-treatment levels and were also significantly higher than those in the other two groups, with all differences being statistically significant (all p < 0.05, Fig. 3A‒B).

### Comparison of IIQ-7 scores and pad test results

Pretreatment assessment revealed no significant differences in 1-hour pad weight or IIQ-7 scores among groups (all p > 0.05). Post-treatment measurements demonstrated significant reductions in both parameters across all groups (all p < 0.01), with the High-Intensity Combined Group showing superior improvement (p < 0.01). Specifically, High-Intensity Combined Group achieved significantly lower post-treatment 1-hour pad weight (2.57±0.44 mL) compared to the Focused PFMT Group (4.67±0.85 mL) and the Generalized PFMT Group (4.56±0.69 mL), along with significantly reduced IIQ-7 scores (7.14±0.87) versus the Focused PFMT Group (11.23±1.29) and the Generalized PFMT Group (11.69±1.32) (Table 3).

**Table 3:** Comparison of 1-hour pad test results and IIQ-7 scores before and after training among the three groups.

Group	1-hour Pad Test (mL)		Within-group p	IIQ-7 Score (points)		Within-group p
	Before Training	After Training		Before Training	After Training	
Focused PFMT Group	8.32 ± 1.75	4.67 ± 0.85	<0.01	16.15 ± 1.72	11.23 ± 1.29	<0.01
Generalized PFMT Group	8.31 ± 1.12	4.56 ± 0.69	<0.01	16.21 ± 1.32	11.69 ± 1.32	<0.01
High-Intensity Combined Group	8.38 ± 1.12	2.57 ± 0.44	<0.01	16.18 ± 1.08	7.14 ± 0.87	<0.01
Between-group p	0.98	<0.01		0.98	<0.01	

Note: Normally distributed continuous data are presented as mean ± standard deviation. Between-group comparisons were performed using two-way analysis of variance with Tukey’s HSD post-hoc test. * p < 0.05 was considered statistically significant.

IIQ-7, Urinary Incontinence Impact Questionnaire.

### Clinical outcomes and recurrence rates

The High-Intensity Combined Group demonstrated a significantly higher overall efficacy rate (87.88%) compared to Focused PFMT Group (60.60%; adjusted p = 0.03) and the Generalized PFMT Group (57.57%; adjusted p = 0.02) (Table 4). At 6-month follow-up, the High-Intensity Combined Group showed lower recurrence rates (15.15%) than the other groups (45.45% and 48.48% in the Focused and Generalized PFMT Groups, respectively), though the difference between the High-Intensity Combined Group and Focused PFMT Group did not reach statistical significance (adjusted p = 0.05) (Table 5).

**Table 4:** Comparison of therapeutic efficacy among the three groups.

Group	N	Cured n (%)	Improved n (%)	Ineffective n (%)	Total Effective n (%)
Focused PFMT Group (A)	33	8 (24.24%)	12 (36.36%)	13 (39.39%)	20 (60.60%)
Generalized PFMT Group (B)	33	8 (24.24%)	11 (33.33%)	14 (42.42%)	19 (57.57%)
High-Intensity Combined Group (C)	33	19 (57.58%)	10 (30.30%)	4 (12.12%)	29 (87.88%)
adj.*P*_Group A/Group B_					1.00
adj.*P*_Group A/Group C_					0.03
adj.*P*_Group B/Group C_					0.02
p-value					0.01

Note: Categorical data are presented as n (%). Overall differences were analyzed using Pearson’s Chi-Square test. When significant differences were found, post-hoc comparisons were performed using Bonferroni correction. Adjusted p-values < 0.05 were considered statistically significant.

**Table 5:** Comparison of recurrence rates at 3- and 6-months post-training among the three groups.

Group	Recurrence n (%)	
	3 Months	6 Months
**Focused PFMT Group (A)**	7 (21.21%)	15 (45.45%)
**Generalized PFMT Group (B)**	9 (27.27%)	16 (48.48%)
**High-Intensity Combined Group (C)**	3 (9.09%)	5 (15.15%)
adj.*P*_Group A/Group B_	1.00	1.00
adj.*P*_Group A/Group C_	0.91	0.05
adj.*P*_Group B/Group C_	0.33	0.02
p-value	0.16	0.01

Note: Categorical data are presented as n (%). Overall differences were analyzed using Pearson’s Chi-Square test. When significant differences were found, post-hoc comparisons were performed using Bonferroni correction. Adjusted p-values < 0.05 were considered statistically significant.

Special Clarification: The adjusted p-value for the comparison of 6-month recurrence rates between the High-Intensity Combined Group and the Focused PFMT Group was 0.047, which rounds to 0.05. As the Bonferroni correction was applied to control the Type I error rate across the three pairwise comparisons (resulting in a corrected significance level of α' = 0.017), a value of 0.047 still exceeds this threshold. Therefore, this result was strictly defined as statistically non-significant.

## Discussion

Pregnancy and childbirth have been identified as independent risk factors for Pelvic Floor Dysfunction (PFD).^17^ PFD occurs due to prolonged overloading and traction of the pelvic floor muscles during pregnancy, with direct compression by the fetal head leading to muscle fatigue and atrophy.^18^ The abrupt pressure increase during delivery causes excessive stretching of pelvic floor muscles, resulting in extreme fiber elongation or even tearing, which may cause irreversible damage to PFM function.^18^ A survey of 1749 primiparous women at 12-months postpartum revealed a 47.5% incidence of SUI.^19^ Studies indicate that most women after vaginal delivery report SUI or other urinary incontinence symptoms, along with vaginal laxity, urinary frequency, and constipation, significantly affecting their quality of life.^20^ This prospective comparative study of three therapeutic regimens (Focused PFMT, Generalized PFMT, and their combination) provides strong evidence for clinical decision-making, demonstrating superior outcomes with the combined approach across multiple parameters.

Generalized PFMT enhances postpartum PFM contractility through sustained training protocols including anal contractions, leg exercises and sit-ups that directly target pelvic floor muscle groups.^21^ However, while Generalized PFMT primarily improves muscular tension, PFM dysfunction often involves concurrent metabolic and oxygenation disturbances.^22^ In contrast, Focused PFMT accelerates neuromuscular recovery in postpartum pelvic floor muscles, promoting long-term functional improvement.^23^ The rhythmic PFM contractions during Focused PFMT increase muscular strength and endurance through enhanced blood circulation and nutrient supply, while simultaneously improving urethral closure pressure via strengthened periurethral muscles.^24^

The high-intensity combined PFMT regimen demonstrated significant advantages in improving pelvic floor function and preventing recurrence. The results showed significantly higher proportions of grade IV‒V muscle strength (approaching 50%) in the High-Intensity Combined Group compared to monotherapy groups. This may be attributed to the intensity effect and the synergistic effect of the combined training modalities. Higher training frequency increases the activation count of pelvic floor muscle fibers, promotes the efficiency of sarcoplasmic reticulum calcium ion cycling, and consequently accelerates the repair and compensatory hyperplasia of damaged muscle fibers. The High-Intensity Combined Group integrated the precise contractions of Focused PFMT with the core support advantages of Generalized PFMT. Focused PFMT, by specifically activating the pubococcygeus muscle, focuses on rapid contractions and enhances the explosive power of the pelvic floor muscles, directly increasing periurethral muscle tension to better support the urethra and maintain its closure during increased abdominal pressure.^25^ This was reflected in the significant increases in MUCP and MUP in urodynamic tests. Generalized PFMT, through actions like anal contractions and sit-ups, strengthens the core muscle groups, helping to enhance the endurance and overall support function of the pelvic floor muscles,^23^ as manifested by optimizations in BC and FUL. The combination of these 2 p.m. forms can also promote blood circulation in the pelvic floor muscles, enhance their metabolic function, and improve BC and detrusor pressure at Qmax. It is noteworthy that no statistically significant differences were found in pelvic floor muscle strength, IIQ-7 scores, or recurrence rates between the Focused PFMT group and the Generalized PFMT group (p > 0.05). This result suggests that merely optimizing the training modality (e.g., focused training), without adequate intensity support, may not enhance the efficacy of PFMT. Although focused Kegel exercises target specific muscle groups, postpartum PFD is often accompanied by decreased coordination among multiple muscle groups (e.g., insufficient synergistic contraction between pelvic floor and abdominal muscles).^22^ Optimization of PFMT training modalities must therefore be based on sufficient training intensity.

Symptomatic improvement represents a critical therapeutic endpoint in SUI management. The combined therapy group showed significantly greater improvement in both the 1-hour pad test leakage volume and the IIQ-7 score compared to the single-therapy groups. Specifically, the 1-hour leakage volume decreased from 8.38±1.12 mL to 2.57±0.44 mL. The significantly lower post-treatment 1-hour leakage volume and IIQ-7 scores in the combined group indicate that combined training can reduce leakage frequency and severity to a greater extent, thereby improving patients’ quality of life. The rationale is that Generalized PFMT can help restore the bladder to its normal physiological position, thereby enhancing bladder tolerance and improving urodynamics. It also strengthens detrusor stability, tolerance to abdominal pressure, and restores pelvic floor muscle strength, alleviating the degree of incontinence. Focused PFMT strengthens perineal muscle force, improves lower urinary tract function, delays bladder overactivity, and increases urethral closure pressure and pelvic floor muscle tension, maintaining detrusor excitability, ultimately improving the clinical symptoms of patients with SUI.

Compared to single-modality training, the advantage of combined training lies in its comprehensiveness. Focused PFMT, while capable of targeting pelvic floor muscles, employs a relatively singular training approach that may not fully cover all parts and functions of the pelvic floor muscles. Generalized PFMT alone, although incorporating various exercises, lacks targeted strengthening of specific pelvic floor muscle functions. High-intensity combined training integrates the advantages of both, strengthening specific pelvic floor muscle functions through Focused PFMT while comprehensively exercising the pelvic floor muscle groups through Generalized PFMT, thereby more effectively enhancing pelvic floor muscle strength. The High-Intensity Combined Group demonstrated significantly higher cure and overall efficacy rates, alongside a significantly lower recurrence rate at the 6-month follow-up.

Therefore, the combination of Focused PFMT and Generalized PFMT holds significant clinical application value and demonstrates good feasibility in treating PSUI. In clinical practice, for patients without exercise limitations and with good adherence, the combined regimen of Focused PFMT and Generalized PFMT is recommended. It is crucial to ensure movement accuracy through high-frequency supervision to prevent compromised form due to high intensity. For patients experiencing significant postpartum fatigue or intolerance to high-intensity training, Generalized PFMT or Focused PFMT alone remain effective options. During treatment, it is necessary to regularly assess muscle strength improvement (e.g., via perineal muscle strength testing every 2-weeks) and patient tolerance. If significant muscle soreness or other discomfort occurs under high-intensity training, the total intensity can be temporarily adjusted individually, resuming gradually after tolerance improves, to prevent decreased adherence due to excessive intensity.

Several study limitations should be acknowledged. The relatively small sample size may affect the generalizability and reliability of the findings. Future studies could expand the sample size and include patients from more diverse regions and with different delivery modes to enhance the general applicability of the results. There is a potential for selection bias. Due to the higher training intensity and the need to master two training forms simultaneously, the screening process for the High-Intensity Combined Group might have preferentially enrolled patients with higher baseline Kegel Exercise Adherence Questionnaire scores (≥12) and stronger subjective willingness to cooperate. This selective enrollment could introduce an adherence bias in the combined group sample, meaning its efficacy advantage might partly stem from baseline adherence differences rather than solely the training regimen’s effect, potentially affecting the external validity of the results. Secondly, the adherence assessment relied on patient self-reporting, which is susceptible to reporting bias. Although an adherence questionnaire was used in this trial, it has not been formally validated, highlighting the need for validated adherence outcome measures. Furthermore, adherence was measured at each visit rather than in real-time, making it subject to recall bias. Additionally, the study duration was relatively short, with follow-ups only at 3- and 6-months, which prevents assessment of the long-term maintenance effect of high-intensity PFMT and fails to record the rate of muscle strength decline after training cessation. Therefore, caution is needed when extrapolating the present findings to long-term efficacy. Subsequent research should extend the follow-up period to observe the long-term impact of combined training on patients, to further validate the long-term efficacy of high-dose combined PFMT in preventing SUI recurrence. Methodological enhancements could include advanced imaging techniques (e.g., magnetic resonance imaging, pelvic ultrasound) for comprehensive structural and functional assessment, along with optimization of training parameters (intensity, frequency, duration) to establish precise clinical guidelines.

The combination of Focused PFMT and Generalized PFMT significantly enhances pelvic floor muscle strength, improves pelvic floor support function and urinary control ability, and reduces the recurrence rate of urinary incontinence symptoms in patients with SUI after vaginal delivery, achieving superior efficacy alongside better long-term outcomes. In summary, training intensity appears to be a key factor in enhancing rehabilitation outcomes. These findings provide a clinical basis for developing comprehensive pelvic floor rehabilitation protocols in clinical practice. For postpartum SUI patients without exercise limitations and with good adherence, the high-intensity combined PFMT regimen is recommended, supplemented by regular muscle strength assessments and follow-up to ensure proper form. For patients with lower postpartum tolerance, single Focused or Generalized PFMT remain effective options, but training intensity should be dynamically adjusted based on individual circumstances to maintain adherence. Future research should further explore the optimal intensity, frequency, and duration of combined training to provide more precise protocol support for postpartum SUI rehabilitation.

## Ethics statement

All procedures performed in this study involving human participants were in accordance with the ethical standards of the institutional and/or national research committee and with the 1964 Helsinki Declaration and its later amendments or comparable ethical standards. All subjects were approved by Zhejiang Shaoxing Shangyu Maternal and Child Health Hospital (No. 202107SX52).

## Authors’ contributions

Conceptualization, YinJing Li; data curation, Chao Jin; formal analysis, YinJing Li and Chao Jin; investigation, YinJing Li; methodology, YinJing Li and Chao Jin; writing-original draft preparation, YinJing Li; writing-review and editing, Chao Jin. All authors have read and agreed to the published version of the manuscript.

## Funding

Not applicable.

## Data availability

Data are available from the First Author on request.

## Declaration of competing interest

The authors declare no conflicts of interest.

## Appendix Supplementary materials

Image, application 1
