# Supplementary material for: Effects of various pelvic floor muscle training forms and combined regimens on muscle strength, function, and recurrence rate in post-vaginal delivery stress urinary incontinence patients
Source: Clinics (Sao Paulo). 2026 Sep 5;81:101088. doi: 10.1016/j.clinsp.2026.101088 (PMC13571952; doi:10.1016/j.clinsp.2026.101088)
Supplement: Supplementary file 1 [file mmc1.docx]

**CLINICS-D-25-01292_ Supplementary Material**

**Supplementary Table 1** PFMT intervention protocol – TIDieR checklist.

| **Item Specific Content** | **Group** | | |
| --- | --- | --- | --- |
| Intervention name | Focused PFMT | Generalized PFMT | High-Intensity Combined |
| Pre-training preparation | Educate patients to help them understand and recognize the pelvic floor muscles; demonstrate and guide under the supervision of a head nurse or above, and monitor the accuracy of patient movements. | | |
| Criteria for starting training | Patient achieves proficiency; visible pelvic floor muscle contraction observed; ultrasound shows upward movement of the bladder base. | | |
| Materials | Audio training version for guided practice. | | |
| Operational guidelines | During inhalation, contract the muscles of the perineum (perineum, anus, urethra) upward. | Anal sphincter exercise: voluntarily contract the pelvic floor muscles without contracting the abdomen, glutes, or lower limbs; Leg exercise: rhythmic and rapid extension and flexion of both legs; Sit-up: perform sit-ups independently, keeping abdominal muscles tightened throughout. | Combined training of Focused PFMT and Generalized PFMT, following the methods, rhythms, and frequencies of each protocol. |
| Exercise position | Supine position, relax whole body, focus attention, breathe deeply and slowly, knees naturally bent, hands placed flat on both sides. | Anal sphincter exercise: supine position, empty bladder before training; Leg exercise: flat supine position; Sit-up: patient in semi-recumbent position with bed at 45°, assist to stabilize legs in hip and knee flexion, feet flat on bed surface, hands in Bobath handshake. |  |
| Contraction differentiation criteria (patient instruction wording) | Two-step guidance: first imagine stopping urine flow (feel the periurethral muscles tighten inward), then add the action of holding back defecation (pull the anus inward and upward). | Imagine holding back defecation to perform anal lifting exercise; feel the perianal skin indent inward; gently place both hands on the abdomen to confirm no movement. |  |
| Contraction-rest interval | Maintain contraction for 5 seconds, relax slowly during exhalation, repeat after 5 seconds. | Rest for 5-minutes between each exercise. |  |
| Intervention frequency | 30-minutes per session, once daily. | Anal sphincter exercise: hold contraction for 5s, relax slowly for 3s, alternate. 10-min/session, once daily; Leg exercise: 10-min/session, once daily; Sit-up: 10-min/session, once daily. |  |
| Home assessment | Patient wears disposable gloves, inserts middle and index fingers into the vagina, and performs an anal contraction. If a sensation of pressure around the fingers is felt, the pelvic floor muscle contraction is appropriate; if no sensation, the contraction is inappropriate. | | |
| Clinical monitoring and supervision measures | Follow-up and reminders via telephone; patient’s caregiver supervises daily completion of exercises; patient records training completion in a logbook. | | |
| Compliance | Patient scores ≥12 on the Kegel Exercise Adherence Questionnaire and completes at least 80% of the prescribed sessions. | | |
| Intervention adjustments | Perform vaginal examination every 2-weeks to adjust movement patterns. | | |
